# Intragraft gene expression profile associated with the induction of tolerance

**DOI:** 10.1186/1471-2172-9-5

**Published:** 2008-02-11

**Authors:** Tomoko Doki, Michael Mello, Dennis Mock, Jacqueline M Evans, Mary Kearns-Jonker

**Affiliations:** 1 Department of Cardiothoracic Surgery Research, Transplantation Biology Research Laboratory, Childrens Hospital Los Angeles, Keck School of Medicine, University of Southern California, Los Angeles, CA, USA; 2 Keck School of Medicine, University of Southern California, Los Angeles, CA, USA; 3 Department of Pathology, USC/CHLA Genome Core, Childrens Hospital Los Angeles, Keck School of Medicine, University of Southern California, Los Angeles, CA, USA; 4 Department of Anesthesiology Critical Care Medicine, Childrens Hospital Los Angeles, Keck School of Medicine, University of Southern California, Los Angeles, CA, USA

## Abstract

**Background:**

Xenotransplantation holds the promise of providing an unlimited supply of donor organs for terminal patients with organ failure. Pre-existing natural antibodies to the Galα1,3Galβ1,4GlcNac-R (αGal) carbohydrate xenoantigen, however, bind rapidly to the graft endothelium and initiate hyperacute rejection of wild type pig grafts in humans. Experimental procedures designed to prevent xenoantibody-mediated rejection have been tested in gal knockout mice. These mice produce anti-gal xenoantibodies and are widely used as small animal models for xenotransplantation research. In this model, chimerism for cells expressing the gal carbohydrate can be achieved by transplantation of mixed cells or by transduction of bone marrow cells with viral vectors expressing a functional α1,3 galactosyltransferase gene. Chimerism induces tolerance to heart grafts expressing αGal. The mechanisms by which tolerance is achieved include systemic changes such as clonal deletion and/or anergy. Intragraft changes that occur during the early stages of tolerance induction have not been characterized.

**Results:**

Cytoprotective genes heme oxygenase-1 (HO-1), Bcl2, and A20 that have been reported to contribute to long-term graft survival in various models of accommodation were not expressed at high levels in tolerant heart grafts. Intragraft gene expression at both early (Day 10) and late (>2 month) time points after heart transplant were examined by real-time PCR and microarray analysis was used to identify changes associated with the induction of tolerance. Intragraft gene expression profiling using microarray analysis demonstrated that genes identified in the functional categories of stress and immunity and signal transduction were significantly up-regulated in early tolerant grafts compared with syngeneic control grafts. Biological process classification showed lower binomial p-values in the categories of "response to biotic stimulus, defense response, and immune response" suggesting that up-regulated genes identified in these grafts promote survival in the presence of an immune response. The expression of the incompatible carbohydrate antigen (αGal) was reduced by 2 months post-transplant when compared with the expression of this gene at Day 10 post-transplant. These results suggest that the gal carbohydrate antigen is downmodulated over time in grafts that demonstrate tolerance.

**Conclusion:**

Our study suggests that tolerance is associated with intragraft gene expression changes that render the heart resistant to immune-mediated rejection. Genes associated with stress and immunity are up-regulated, however cytoprotective genes HO-1, Bcl2 and A20 were not up-regulated. The expression of the gal carbohydrate, the key target initiating an immune response in this model, is down-regulated in the post-transplant period.

## Background

The use of pigs as organ donors could potentially provide an unlimited supply of organs for patients with end-stage organ failure. The Galα1,3Galβ1,4GlcNac-R (αGal) carbohydrate expressed on wild type pig organs, however, initiates the rapid rejection of these grafts [1]. The α1,3 galactosyltransferase (GalT) knockout model (GalT^-/-^) in mice provides a unique system in which to study the immunological events associated with the rejection of cells or organs expressing the gal carbohydrate [2]. Several promising therapies designed to prevent graft rejection have been studied in this model, including the induction of chimerism to achieve transplant tolerance [3]. Mixed chimerism, acquired by transplantation of the donor's bone marrow cells into the recipient, results in tolerance to xenoreactive T cells as well as B cells [4]. Molecular chimerism, acquired by transplantation of transduced, autologous cells expressing a new gene has also been successfully applied to achieve tolerance [5]. Our group has focused on the use of gene therapy using lentiviral vectors to express the porcine α1,3 galactosyltransferase gene and establish a state of chimerism as a means of achieving transplant tolerance [6-8]. Irrespective of the methodology applied to establish chimerism prior to transplantation, receptor editing and/or clonal deletion play a role in the induction of tolerance [7,9,10]. In accommodation models, in which a transplanted organ may survive continuously in the presence of anti-graft antibodies and complement that might otherwise cause rejection, systemic events as well as intragraft gene expression changes have been shown to contribute to prolonged graft survival [11]. Cytoprotective genes are induced during accommodation and protect the grafts by blocking the activation of nuclear factor kappa B (NF-κB) and preventing apoptosis [12,13]. Intragraft gene expression changes associated with the induction of transplant tolerance are less well-characterized and may differ between models [14-18]. The development of gene expression profiling using microarrays has now provided a technologically sophisticated means of studying intragraft gene expression profiles in tolerant and/or rejected grafts [19-25]. Identification of distinct patterns of gene expression changes in graft biopsies may be useful in predicting graft outcome.

In this manuscript, early intragraft gene expression changes associated with the induction of chimerism and tolerance are identified. We demonstrate that expression of cytoprotective genes, heme oxygenase-1 (HO-1), Bcl2, and A20, do not play a role in tolerance induction in this model. This new information can be used to compare early gene expression profiles associated with various models of tolerance induction with the goal of identifying common intragraft gene expression changes that promote graft survival.

## Results

### The level of GalT expression in transduced bone marrow cells

**Figure 1:**
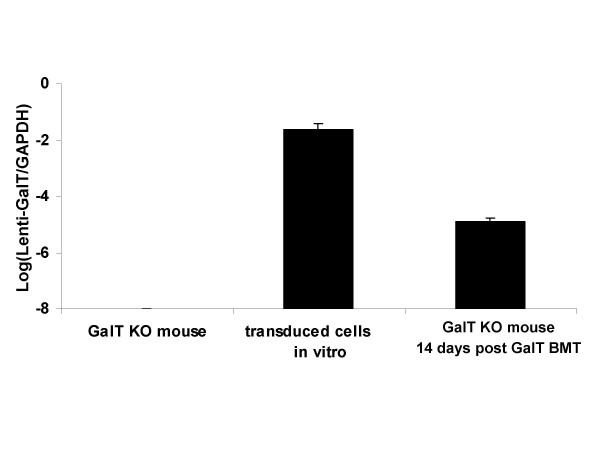
**Level of GalT transduction in bone marrow cells**. GalT is not expressed in the bone marrow cells of the normal GalT^-/- ^mouse, however, transduced bone marrow cells *in vitro *and bone marrow cells from GalT BMT mice at day 14 post-BMT express the galactosyltransferase gene as identified by real-time PCR. Relative cDNA expression levels were normalized with respect to GAPDH gene expression as an internal control. The samples were run in triplicate and the experiment was repeated twice. Standard deviations are shown for each experiment.

Sublethally irradiated GalT^-/- ^mice transplanted with transduced bone marrow expressing GalT prior to heart transplantation demonstrated chimerism and permanently accepted heart grafts from wild type mice, consistent with data previously reported from our laboratory [7]. The level of GalT transduction in bone marrow cells was examined by real-time PCR using primers that specifically identify the galactosyltransferase gene in the lentiviral vector construct. The newly-introduced galactosyltransferase gene was expressed in transduced bone marrow cells *in vitro *as well as *in vivo *in bone marrow cells isolated from GalT^-/-^mice at fourteen days post-GalT bone marrow transplantation (BMT) as identified by real-time PCR (Fig. 1).

### Cytoprotective genes

**Figure 2:**
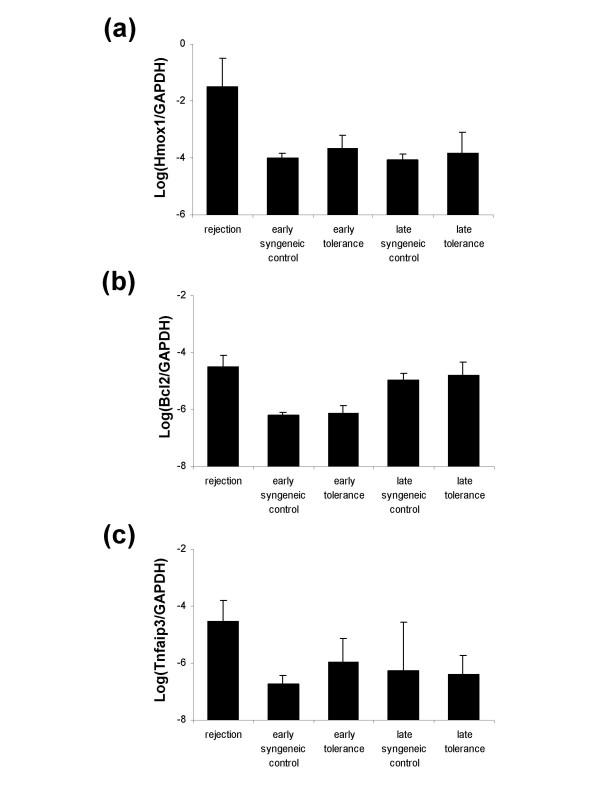
**Cytoprotective gene expression by real-time PCR**. Cytoprotective genes (Hmox1, Bcl2, Tnfaip3) expressed in syngeneic control grafts and in tolerant grafts at both early (day 10) and late (>2 months) time points after transplantation were identified by real-time PCR. Relative cDNA expression levels were normalized with respect to GAPDH gene expression as an internal control. Results are shown as the logarithmic value of respective gene expression. Standard deviations are shown for each experiment which was run in triplicate. (a): Hmox1 (HO-1); (b): Bcl2; (c): Tnfaip3 (A20).

Genes such as heme oxygenase-1 (HO-1; Hmox1, Hsp32), Bcl2, and A20 (Tnfaip3) have been associated with accommodation in rodent xenograft models [11-13]. In contrast, gene expression changes studied in biopsies of accommodated kidney allografts in humans failed to demonstrate significant changes in the expression levels of the same cytoprotective genes [26]. To investigate whether cytoprotective genes are expressed in tolerant grafts in our model, we compared the expression levels of HO-1, Bcl2 and A20 by real-time PCR in transplanted tolerant and syngeneic control hearts. Total RNA was isolated from grafts at day 10 post-heart transplantation. The selection of this time point allowed us to study gene expression changes that occur in graft hearts at the early stages of tolerance induction. We also isolated total RNA from grafts in GalT BMT chimeras demonstrating long-term tolerance to αGal^+ ^heart grafts during later stages of tolerance (>2 months post-heart transplantation) to allow us to compare levels of cytoprotective gene expression at early and later time points. Lymphocyte infiltration that was evident in rejecting grafts could not be detected in tolerant hearts as identified by histology at both early and late time points after transplantation. Infiltrating immune cells were therefore not likely to contribute substantially to gene expression changes in the tolerance model. Our results show that cytoprotective genes were not increased at either early or late time points in tolerant grafts (Fig. 2a–c). These data suggest that the mechanism of tolerance in this model does not involve selective up-regulation of these genes.

### Gene expression profiling in tolerant heart grafts: Functional classification of significantly up-regulated genes

**Figure 3:**
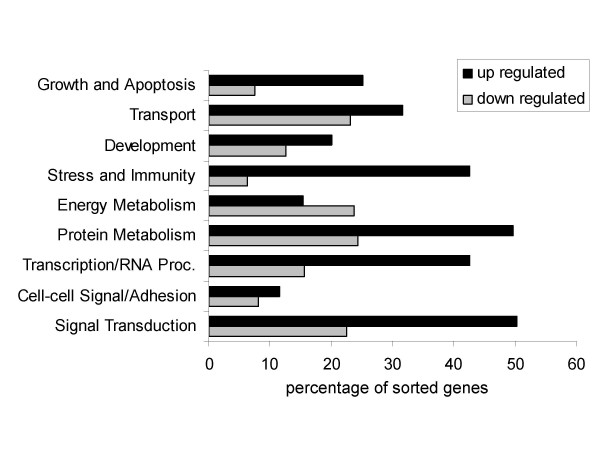
**Functional subsets that are up- or down-regulated in tolerance model**. L2L software was used to sort the up- or down-regulated genes in tolerant grafts (GalT BMT) compared to syngeneic control grafts. Nine functional subsets were identified using Gene Ontology Biological Process categories.

In order to further understand the early events that occur within tolerant grafts, we extended our analysis to the application of gene expression profiling. This technique makes it possible to identify novel gene expression changes that characterize the immunological events associated with tolerance induction. We isolated RNA from transplanted hearts following GalT BMT at ten days after heart transplantation (n = 4) as well as from syngeneic heart transplant controls (n = 4) for microarray analysis. Our objective was to identify novel genes and pathways that may be associated with the early stages of tolerance induction and to compare our data with information in the literature identifying genes uniquely expressed in tolerant grafts. The data were normalized with the dChip software and analyzed by the algorithm "Significant Analysis of Microarrays (SAM)" with a q-value cutoff of 10% and a fold change of greater than 1.5 and less than 0.75. Using these criteria, 535 probe sets representing 465 genes were selected as up-regulated genes and 311 probe sets representing 272 genes were selected as down-regulated. In order to determine whether these genes could be associated with specific functional categories, we used L2L software (ver. 2006.2) to sort the data into each of 9 functional subsets according to the Gene Ontology (GO; ver. 2006.2) "biological process" categories (Fig. 3). The genes classified in the subsets of "stress and immunity" (GO: 0006955, 0006350), "transcription/RNA processing" (GO: 0007165), and "signal transduction" (GO: 0016070, 0006350) were significantly up-regulated during the early stages of tolerance induction. Genes classified in the "energy metabolism" (GO: 0005975, 0006118, 0006629, 0006119, 0006800) category were down-regulated.

### Profiling of the biological processes associated with significantly up-regulated genes induced in tolerant heart grafts

L2L software was used to sort and prepare a summary profile of biological processes associated with genes with increased expression in tolerant grafts as compared to controls at the day 10 time point (Table 1). This table separates genes according to biological processes using a p-value of less than 0.01 as a basis for selection. Genes are ranked according to "binomial p-values," the p-values representing the statistical significance of the overlap, derived from a binomial distribution. "Total probes" signifies the total number of probes representing genes on the list. The list of "expected matches" includes the number of probes derived from the list that would match our data by random chance. The list of "actual matches" shows the number of matches identified with our data. "Fold enrichment" is the fold-enrichment of genes that match our data (actual/expected). As shown in Table 1, the top three categories for function of genes with increased expression in tolerant hearts were "response to biotic stimulus", "defense response" and "immune response." Gene Ontology defines "response to biotic stimulus" as a change in state or activity of a cell or an organism (in terms of movement, secretion, enzyme production, gene expression, etc.) as a result of a biotic stimulus, a stimulus caused or produced by a living organism. The genes in this category show a 2.7 fold enrichment, the highest number of actual matches, and the lowest binomial p-values. Genes in the "defense response" and "immune response" categories demonstrated similar levels of fold enrichment, actual matches and low binomial p-values. Within these 3 categories, fifty-five up-regulated genes that were common to all three categories were identified as preferentially expressed during the early stages of tolerance induction in our model (Table 2).

**Table 1:** Gene Ontology: biological process – up-regulated genes in tolerant hearts compared to syngeneic controls

List Name	Total probes	Expected matches	Actual matches	Fold Enrichment	Binomial p-values
response to biotic stimulus	1353	22.64	61	2.69	1.16E-11
defense response	1275	21.33	53	2.48	3.86E-09
immune response	1166	19.51	50	2.56	3.87E-09
response to virus	108	1.81	12	6.64	3.04E-07
response to pest, pathogen or parasite	767	12.83	33	2.57	1.41E-06
response to other organism	782	13.08	33	2.52	2.13E-06
response to stimulus	3134	52.44	87	1.66	6.47E-06
JAK-STAT cascade	66	1.1	8	7.24	1.49E-05
protein kinase cascade	610	10.21	26	2.55	2.02E-05
response to pathogenic bacteria	24	0.4	5	12.45	4.27E-05
regulation of DNA binding	15	0.25	4	15.94	9.23E-05
I-kappaB kinase/NF-kappaB cascade	223	3.73	13	3.48	1.18E-04
nuclear transport	254	4.25	14	3.29	1.19E-04
nucleocytoplasmic transport	276	4.62	14	3.03	2.79E-04
response to pathogen	35	0.59	5	8.54	2.80E-04
maintenance of localization	38	0.64	5	7.86	4.15E-04
response to bacteria	59	0.99	6	6.08	4.63E-04
regulation of binding	23	0.38	4	10.39	5.38E-04
response to stress	1778	29.75	49	1.65	6.71E-04
T cell receptor signaling pathway	14	0.23	3	12.81	1.48E-03
immune cell mediated cytotoxicity	15	0.25	3	11.95	1.83E-03
negative regulation of protein import into nucleus	15	0.25	3	11.95	1.83E-03
negative regulation of transcription factor import into nucleus	15	0.25	3	11.95	1.83E-03
cytoplasmic sequestering of transcription factor	15	0.25	3	11.95	1.83E-03
cytoplasmic sequestering of protein	15	0.25	3	11.95	1.83E-03
negative regulation of nucleocytoplasmic transport	16	0.27	3	11.21	2.23E-03
negative regulation of protein transport	16	0.27	3	11.21	2.23E-03
inflammatory response	311	5.2	13	2.5	2.57E-03
cholesterol metabolism	140	2.34	8	3.42	2.61E-03
tyrosine phosphorylation of STAT protein	17	0.28	3	10.55	2.67E-03
regulation of NF-kappaB import into nucleus	17	0.28	3	10.55	2.67E-03
NF-kappaB import into nucleus	17	0.28	3	10.55	2.67E-03
antigen receptor-mediated signaling pathway	18	0.3	3	9.96	3.17E-03
nuclear export	115	1.92	7	3.64	3.34E-03
cell killing	19	0.32	3	9.44	3.71E-03
sterol metabolism	149	2.49	8	3.21	3.82E-03
detection of stimulus	63	1.05	5	4.74	4.12E-03
positive regulation of apoptosis	368	6.16	14	2.27	4.18E-03
positive regulation of programmed cell death	368	6.16	14	2.27	4.18E-03
detection of external stimulus	41	0.69	4	5.83	4.85E-03
detection of abiotic stimulus	41	0.69	4	5.83	4.85E-03
DNA metabolism	1205	20.16	33	1.64	4.94E-03
viral genome replication	42	0.7	4	5.69	5.29E-03
RNA export from nucleus	97	1.62	6	3.7	5.94E-03
nucleic acid transport	97	1.62	6	3.7	5.94E-03
RNA transport	97	1.62	6	3.7	5.94E-03
establishment of RNA localization	97	1.62	6	3.7	5.94E-03
caspase activation	44	0.74	4	5.43	6.25E-03
response to drug	44	0.74	4	5.43	6.25E-03
positive regulation of caspase activity	44	0.74	4	5.43	6.25E-03
lipid transport	131	2.19	7	3.19	6.78E-03
RNA localization	101	1.69	6	3.55	7.20E-03

**Table 2:** Up-regulated genes in tolerant hearts classified as *response to biotic stimulus*, *defense response*, and *immune response*

Probe ID	Gene Symbol	Gene Title	Also known as
1416111_at	Cd83	CD83 antigen	
1416295_a_at	Il2rg	interleukin 2 receptor, gamma chain	CD132
1416697_at	Dpp4	dipeptidylpeptidase 4	Cd26; THAM; Dpp-4
1459973_x_at	Dpp4		
1417056_at	Psme1	proteasome (prosome, macropain) 28 subunit, alpha	PA28a
1417189_at	Psme2	proteasome (prosome, macropain) 28 subunit, beta	PA28b
1417640_at	Cd79b	CD79B antigen	B29; Igb; Ig-beta
1418652_at	Cxcl9	chemokine (C-X-C motif) ligand 9	CMK; Mig; Scyb9; crg-10
1419282_at	Ccl12	chemokine (C-C motif) ligand 12	MCP-5; Scya12
1419684_at	Ccl8	chemokine (C-C motif) ligand 8	HC14; MCP-2; Scya8
1420089_at	Nfkbia	nuclear factor of kappa light chain gene enhancer in B-cells inhibitor, alpha	
1448306_at	Nfkbia		
1449731_s_at	Nfkbia		
1420788_at	Klrg1	killer cell lectin-like receptor subfamily G, member 1	MAFA; 2F1-Ag; MAFA-L
1420915_at	Stat1	signal transducer and activator of transcription 1	
1450033_a_at	Stat1		
1450034_at	Stat1		
1421578_at	Ccl4	chemokine (C-C motif) ligand 4	Act-2; Mip1b; Scya4; MIP-1B
1421818_at	Bcl6	B-cell leukemia/lymphoma 6	Bcl5
1421911_at	Stat2	signal transducer and activator of transcription 2	
1422005_at	Eif2ak2	eukaryotic translation initiation factor 2-alpha kinase 2	Pkr; tik; Prkr
1422028_a_at	Ets1	E26 avian leukemia oncogene 1, 5' domain	Tpl1; Ets-1
1422903_at	Ly86	lymphocyte antigen 86	MD1
1422962_a_at	Psmb8	proteosome (prosome, macropain) subunit, beta type 8 (large multifunctional peptidase 7)	Lmp7
1424208_at	Ptger4	prostaglandin E receptor 4 (subtype EP4)	EP4; Ptgerep4
1425396_a_at	Lck	lymphocyte protein tyrosine kinase	Hck-3; p56
1425548_a_at	Lst1	leukocyte specific transcript 1	B144
1426587_a_at	Stat3	signal transducer and activator of transcription 3	Aprf
1427689_a_at	Tnip1	TNFAIP3 interacting protein 1	Nef; ABIN; Naf; ABIN1
1427746_x_at	H2-K1	histocompatibility 2, K1, K region	H-2K; H2-K; MHC I;
1429272_a_at	Apol3	apolipoprotein L 3	
1433508_at	Klf6	Kruppel-like factor 6	FM2; FM6; Zf9; BCD1; CPBP; Copcb; Ierepo1; Ierepo3
1434438_at	Samhd1	SAM domain and HD domain, 1	Mg11
1435560_at	Itgal	integrin alpha L	Cd11a; LFA-1; Ly-15; Ly-21
1435710_at	Cd226	CD226 antigen	Pta1; PNAM1; TLiSA1
1435906_x_at	Gbp2	guanylate nucleotide binding protein 2	
1436562_at	Ddx58	DEAD (Asp-Glu-Ala-Asp) box polypeptide 58	RIG-I
1436779_at	Cybb	cytochrome b-245, beta polypeptide	Cgd; Nox2; gp91phox
1437304_at	Cblb	Casitas B-lineage lymphoma b	
1438052_at	Ptprc	protein tyrosine phosphatase, receptor type, C	Ioc; Ly-5; T200; CD45R; Lyt-4
1439034_at	Spn	sialophorin	Cd43; Ly48; Galgp
1439680_at	Tnfsf10	tumor necrosis factor (ligand) superfamily, member 10	TL2; Ly81; Trail; APO-2L
1439773_at	Ly6e	lymphocyte antigen 6 complex, locus E	Ly67; Tsa1; RIG-E; Sca-2; TSA-1
1439819_at	Ctsc	cathepsin C	DPP1
1440169_x_at	Ifnar2	interferon (alpha and beta) receptor 2	
1441026_at	Parp4	poly (ADP-ribose) polymerase family, member 4	PH5P; p193; Gm743; PARPL; VPARP; VAULT3; Adprtl1
1445897_s_at	Ifi35	interferon-induced protein 35	IFP35
1459151_x_at	Ifi35		
1448436_a_at	Irf1	interferon regulatory factor 1	

### Intragraft α1,3 galactosyltransferase gene expression

**Figure 4:**
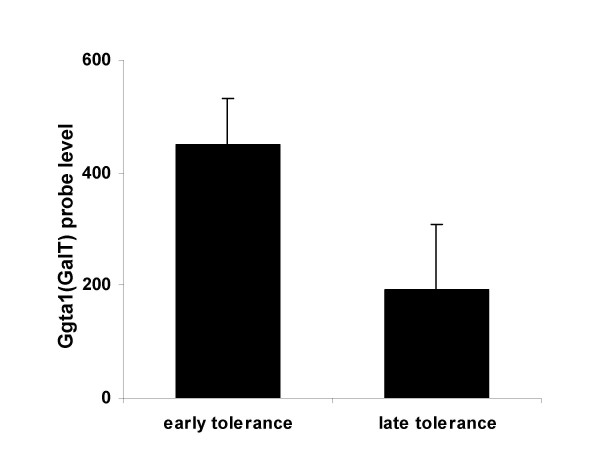
**Intragraft GalT expression changes in tolerant heart grafts**. Ggta1 (1418483_a_at) probe expression levels at early (day 10) and late (>2 months) time points in tolerant hearts, identified by microarray analysis. Standard deviations are shown as error bars.

An additional factor that plays a key role in long-term graft survival is the level of expression of xenoantigens on the surface of the graft. Accommodation has been associated with a decrease in the expression level of incompatible carbohydrate antigens on highly vascularized grafts exposed to tolerable levels of allo- or xenoantibodies [27]. We therefore evaluated whether or not changes in expression level of the gene encoding for the gal carbohydrate occur in either the immediate or late post-transplant periods in our model of tolerance induction. Expression of the galactosyltransferase gene which encodes the target of rejection of wild type organs in this model was significantly reduced during the late post-transplant period (Fig. 4), suggesting that a reduction in the level of expression of the αGal epitope occurs on the graft at later time points post transplant. Lower levels of expression of the gal carbohydrate may therefore contribute to the maintenance of long-term tolerance in this model.

### Validation of microarray results by real-time PCR

**Figure 5:**
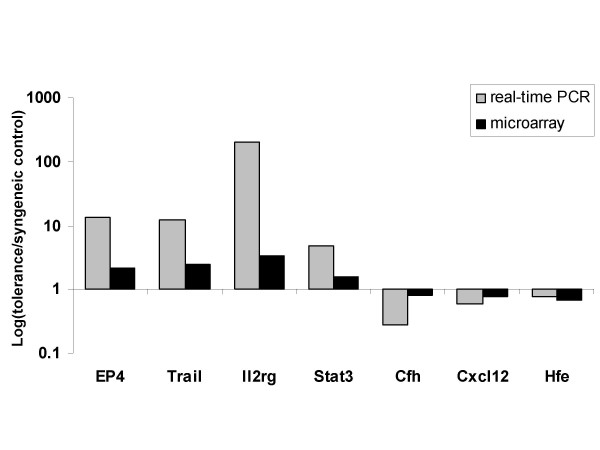
**Validation of microarray results by real-time PCR**. Selected genes (Ptger4, Tnsls10, Il2rg, Stat3, Cfh, Cxcl12, Hfe) in the early tolerance group (GalT BMT) compared to syngeneic controls were analyzed by real-time PCR to determine whether the data obtained by microarray analysis could be validated using an alternative technique. Relative cDNA expression levels were normalized with respect to GAPDH gene expression as internal control. Results are shown as the logarithmic value of mean fold-change of gene expression.

Real-time PCR was used to confirm the results obtained from the gene expression profiling studies. Four up-regulated genes and 3 down-regulated genes were selected for quantification of gene expression by real-time PCR. RNA from transplanted hearts isolated from GalT BMT groups (n = 4) and syngeneic control groups (n = 4) was used for this experiment. Real-time PCR results were found to correlate with the differential gene expression data obtained by the microarray analysis (Fig. 5). The sequences of primers used for real-time PCR validation are listed in Table 3.

**Table 3:** Primer sequences used to identify gene transcript levels by real-time PCR

Gene Symbol		Sequence (5' -> 3')
lenti-porcine GalT	sense	GTT CGC TTC TCG CTT CTG TT
	antisense	CCA AAA CAC AAC CAT TAC AGT TGA G
Ptger4 (EP4)	sense	TAC TTC TAC AGC CAC TAC GTG GAC
	antisense	TGG TCC AGT CGA TGA AGC ACC AGG
Tnfsf10 (Trail)	sense	ACC ACG TGC TCT TTA GGA ATG GAG
	antisense	AGA CCA TCT TGG AAG CGT CTT CAG
Il2rg (CD132)	sense	GGT TGG AAC GAA TGC CTC CAA TTC
	antisense	GCA GAA CCG TTC ACT GTA GTC TGG
Stat3	sense	GCA AAG AGT CAC ATG CCA CGT TGG
	antisense	AGA TAC CTG CTC TGC AGA AAC TGC
Cfh	sense	AAG GTG GCA GTC ATT ACC TCG CTG
	antisense	GTT CAT GAC TGC TGG ACT CAA TGG
Cxcl12	sense	CGC TCT GCA TCA GTG ACG GTA AAC
	antisense	CTT CAG CCG TGC AAC AAT CTG AAG
Hfe	sense	TCT CTA AGG TGT CAG GCT CTG GAC
	antisense	TGT CAG CCA GCC TTG ATA GGT CTC
Foxp3	sense	TCC AGA GAG AAG TGG TGC AGT CTC
	antisense	GTG GCT ACG ATG CAG CAA GAG CTC
Hmox1 (HO-1)	sense	ACA GAT GGC GTC ACT TCG TCA GAG
	antisense	ACT GCC ACT GTT GCC AAC AGG AAG
Bcl2	sense	GAT GCC TTT GTG GAA CTA TAT GGC
	antisense	AGG TAT GCA CCC AGA GTG ATG CAG
Tnfaip3 (A20)	sense	CTA AGC CAA CGA GTA GGT TCT GTG
	antisense	CCA TAC ATC TGC TTG AAC TGG TAG
GAPDH	sense	GGC ATG GAC TGT GGT CAT GAG
	antisense	TGC ACC ACC AAC TGC TTA GCC

## Discussion

An understanding of the systemic and intragraft gene expression changes that promote immune modulation and long term graft survival may provide insight into new ways to achieve tolerance. One of the most well-studied methods for inducing tolerance is by the induction of chimerism in Gal KO mice [3-7]. These mice do not express the galactosyltransferase that is responsible for gal carbohydrate expression and produce high levels of anti-gal antibodies after immunization [28]. As gal knockout mice age, they naturally produce anti-α Gal antibodies at levels that are sufficient to cause delayed rejection of wild type mouse hearts [29]. Anti-gal xenoantibodies induced after immunization are capable of initiating hyperacute rejection of gal^+ ^hearts [28]. This small animal model is extensively used to study xenoantibodies to grafts that express the gal carbohydrate, the major antigen responsible for initiating xenograft rejection. Chimerism for the gal carbohydrate can be achieved in a number of ways and results in tolerance to wild type gal^+ ^heart grafts [3-7].

The mechanisms by which tolerance can be achieved after the induction of chimerism include systemic changes such as B cell hyporesponsiveness, initially due to anergy, and receptor editing or clonal deletion which occur later [9]. Regulatory T cells can also contribute to long-term graft survival [30]. Our laboratory has been interested in studying the induction of chimerism using lentiviral vectors to express the galactosyltransferase gene in the bone marrow. In this model, systemic events associated with tolerance induction similarly include receptor editing and/or clonal deletion [7]. Cytoprotective IgG2b antibodies [7] and elevated levels of cytoprotective genes HO-1 and A20 that contribute to graft accommodation [11,31] were not induced at levels higher than those identified in syngeneic transplant controls [21]. Data similar to ours was recently reported in a clinical study which showed that protective genes Bcl-2 and A20 were not induced in human cardiac biopsies taken at 0–2 months from non-rejecting grafts, however these genes were shown to be induced at later times (>10 months post-transplant) [32]. The time between transplantation and gene expression analysis may therefore be an important consideration. Cytoprotective genes were, however, induced at early timepoints in the rejecting grafts in our study. This finding is consistent with prior reports that A20 and/or HO-1 are upregulated in response to immune injury during acute rejection of renal, heart and lung allografts [32-34]. These gene products have anti-inflammatory and anti-apoptotic functions which may be produced in an attempt to protect grafts from injury [35-37]. Expression of the gene encoding the gal carbohydrate, in contrast, was significantly reduced in tolerant hearts. Similar findings have been reported in models of accommodation where expression of endothelial carbohydrate antigens declines post-transplantation [27]. The level of expression of the gal carbohydrate influences not only the antibody response, but impacts the cellular immune response. NK cells have receptors that recognize the α Gal epitope, and this recognition may contribute to the induction of a cellular immune response in the post-transplant period [38,39].

The gene expression profile associated with tolerance induction may be a key predictor of graft outcome. Within the past few years, microarray technology has been applied to identify gene expression changes that distinguish tolerance and rejection in various models. Although the conditions under which tolerance is induced are not uniform, the source of cells or tissues varies, the biostatistical analysis differs and the software used in studies from other laboratories varies, differential gene expression in stress-activated pathways and immune response genes tends to distinguish tolerant grafts from controls [21,22,40-43]. Our results are strikingly similar to those reported from two other laboratories who identified a small number of genes that were associated with the induction and maintenance of tolerance to liver grafts in rodents. The genes that were common to the molecular signature of tolerance in these three studies included STAT-1, IRF-1, Gpb2 and several chemokines [42,43]. STAT-1 and IRF-1 are the two key transcripts that play a role in the pathway that links IFN-γ signalling to the induction of apoptosis [44,45]. The fact that these transcripts were induced in the early stages of tolerance to heart and liver grafts [42] and in the PBL of tolerant recipients of liver grafts at 100 days post-transplantation [43] suggests that the STAT-1/IRF-1 apoptotic pathway may be important in the induction and maintenance of graft tolerance. The data from our lab and others suggests that the tolerant graft itself responds on a continuous basis to the environment around it.

Tolerance to pig organs is a long-term goal in the field of xenotransplantation. Whether this is achieved by introduction of new genes in the donor organs prior to transplantation, or by the development of methods that adequately suppress the immune response to porcine xenoantigens, gene expression profiling of tolerant grafts is providing new insight into the mechanisms by which tolerance is achieved and maintained. An improved understanding of the similarities and differences in intragraft gene expression profiles that are associated with tolerance induced by various approaches and a clear statement of the methods used for the analysis of the data should allow new opportunities to identify common parameters in intragraft gene expression profiles in tolerant grafts. In addition, the development of accurate, targeted and reliable tests for gene expression in graft biopsies may someday be available for use in clinical transplantation as a means to monitor graft survival.

## Conclusion

In this study, we have described the intragraft gene expression changes that associate with the induction of tolerance to the αGal carbohydrate. Although the cytoprotective genes HO-1, Bcl2 and A20 were not induced in the early stages of tolerance induction in this model, genes associated with stress and immunity were up-regulated. This result suggests that the tolerant graft itself responds on a continuous basis to the environment around it. The reduction of αGal carbohydrate gene expression over time in tolerant grafts indicates the possibility of adaptation of the graft. An improved understanding of the similarities and differences in intragraft gene expression profiles that are associated with tolerance induced by various approaches and a clear statement of the methods used for the analysis of the data should allow new opportunities to identify common parameters in intragraft gene expression profiles in these grafts.

## Methods

### Mice

Gal T^-/- ^mice homozygous for the targeted disruption in the GalT gene do not express the αGal epitope and produce anti-αGal-reactive antibodies in their serum. GalT^-/- ^mice were backcrossed 10 times using C57BL/6 mice (Jackson Laboratory, Bar Harbor, ME) and were obtained from Dr. A. d'Apice (St Vincent's Hospital in Melbourne, Australia). The mice used for these experiments were 12 to 16 weeks age at the time of heart transplantation. All animals received humane care in compliance with the Principles of Laboratory Animal Care, formulated by the National Society for Medical Research, and the Guide for the Care and Use of Laboratory Animals, prepared by the National Institutes of Health.

### Transplantation of bone marrow cells transduced by lentiviral vectors

A nonmyeloablative regimen was used to establish chimerism by transplantation of bone marrow cells transduced with a lentiviral vector expressing porcine α1,3 galactosyltransferase, as previously described [7]. Briefly, bone marrow cells were flushed from the femurs of GalT^-/- ^mice and were transduced with a lentiviral vector expressing the porcine α1,3 GalT gene. Recipient mice were matched for age and anti-αGal Ab levels prior to bone marrow transplantation (BMT). Mice were sublethally irradiated with 3 Gy of whole-body irradiation using a ^137^Cs irradiator. Transduced bone marrow cells (2.7 × 10^7 ^to 5.0 × 10^7 ^cells) were administered by tail vein injection to recipient GalT^-/- ^mice within 48 hours after irradiation.

### Heterotopic heart transplantation

Intra-abdominal heterotopic heart transplantation was performed at 2 to 4 weeks after BMT [46]. Heart grafts from C57BL/6 mice were transplanted into GalT BMT mice (n = 6), C57BL/6 syngeneic control mice (n = 8), and GalT^-/- ^mice that did not receive a BMT to induce chimerism (n = 6). Mice were anesthetized with 1.5 to 2.0% Isoflurane. The heart grafts from C57BL/6 mice which were transplanted to GalT^-/- ^mice in the absence of a BMT to achieve chimerism were rejected at an average of 12.8 days after transplantation. Graft function was monitored daily by palpation. At day 10 post heart transplantation, recipient mice were euthanized for examination. Tolerance at early and late time points was compared by isolation of hearts at Day 10 (n = 6) and at >2 months post-heart transplantation (n = 3). Matched syngeneic control mice were examined at early and late time points (n = 8, n = 3 each). The RNA was extracted from the graft tissue and microarray data were used to compare gene expression at the early and later time points.

### RNA extraction from heart graft samples

The graft heart tissues for RNA extraction were immediately frozen and kept at -80°C. RNA was isolated and purified using an RNeasy Fibrous Tissue Mini Kit (Qiagen, Valencia, CA) according to manufacturer's instructions. Optical density was measured by spectrophotometry at 260 and 280 nm, and integrity of total RNA was confirmed by agarose gel electrophoresis.

### Microarray target preparation and hybridization for Affymetrix GeneChips

Affymetrix GeneChip Mouse 430 2.0 Expression Arrays were used for this study. Two μg of tRNA was reverse transcribed using a T7-Oligo(dT) primer. Second-strand cDNA was purified and used as a template for *in vitro *transcription (IVT). IVT with T7 RNA Polymerase and biotin yielded labeled cRNA targets that were then fragmented and 10 μg hybridized to the GeneChip. These procedures were performed by the USC/CHLA Genome Core, Department of Pathology, Childrens Hospital Los Angeles according to the manufacturer's protocol (Affymetrix, Santa Clara, CA).

### Microarray data analysis

The processed image file of the Affymetrix Mouse 430v2 array contained over 45000 probe sets covering approximately 39000/34000 transcripts/genes [47]. The probe set level data were analyzed with the dChip software [48]. Values deemed "present' using the PM/MM correction model with single probe rejection were considered further. The algorithm "Significant Analysis of Microarrays (SAM)" which uses a permutation test to set the "no-change" expression threshold, selecting genes that showed statistically significant differences for each condition. The program generated a false positive rate or a q-value for each gene for the replicated samples using a nearest neighbor metric. This test creates a set of individual genes that are differentially expressed for each ligand-time condition. To note the enrichment of a particular gene annotation from the list of differentially expressed genes determined to be statistically significant (p < 0.01), we used the hypergeometric distribution formula. The gene ontology was obtained from the L2L database [49]. We selected the gene attribute "biological process" as described by the Gene Ontology Consortium [50] for subgroup enrichment. We elected to use dChip, SAM, and L2L software packages as they are available free of charge to facilitate the comparison of our data with that obtained by other investigators interested in the identification of genes expressed in tolerant grafts. The microarray data used in this study was deposited in the National Center for Biotechnology Information (NCBI) Gene Expression Omnibus (GEO) [51] with accession numbers GSM179880 through GSM 179573. The microarray data are also available in a series with accession number GSE 7424.

### Quantitative real-time PCR

Total RNA was reverse-transcribed into cDNA (Omniscript RT Kit, Qiagen, Valencia, CA). Quantitative real-time PCR was performed using an ABI PRISM 7700 Sequence Detector (Perkin Elmer, Foster City, CA) and a Quantitect SYBR Green PCR Kit (Qiagen, Valencia, CA) according to manufacturer's protocol. PCR amplification was performed at 95°C for 15 min followed by 45 cycles of 95°C for 15 sec, 56°C for 30 sec, and 72°C for 30 sec. Each PCR was performed in triplicate. Samples were electrophoresed on a 1.5% agarose gel to confirm that nonspecific amplification did not occur. Results were expressed relative to the housekeeping gene, glyceraldehyde-3-phosphate dehydrogenase (GAPDH). Oligonucleotides used as primers in this study are shown in Table 3.

## List of abbreviations

All abbreviations are defined at their first appearance in the text, and in the legends of tables and figures, as follows:

αGal – Galα1,3Galβ1,4GlcNac-R

GalT – α1,3 galactosyltransferase

GalT^-/- ^– gal knockout

NF-κB – nuclearfactor kappa B

BMT – bone marrow transplantation

HO-1 – heme oxygenase-1

SAM – significant analysis of microarrays

Gene Ontology – GO

IVT – *in vitro *transcription

NCBI – National Center for Biotechnology Information

GEO – Gene Expression Omnibus

## Authors' contributions

TD performed the heart transplants, prepared the RNA, was responsible for the real-time PCR and the microarray data analysis, and drafted the manuscript. MM transduced the bone marrow cells and produced the bone marrow chimeras. DM analyzed the gene expression arrays and provided suggestions for the statistical analysis of the microarray data. JME provided the RNA for the PCR used to identify gene expression at >2 months post-transplant. MKJ designed the study, participated in its coordination, and made major contributions to the manuscript. All authors read and approved the final manuscript.
